# Functional Genomic Analysis of Variation on Beef Tenderness Induced by Acute Stress in Angus Cattle

**DOI:** 10.1155/2012/756284

**Published:** 2012-04-12

**Authors:** Chunping Zhao, Fei Tian, Ying Yu, Juan Luo, Apratim Mitra, Fei Zhan, Yali Hou, George Liu, Linsen Zan, M. Scott Updike, Jiuzhou Song

**Affiliations:** ^1^College of Animal Science and Technology, Northwest A&F University, Yangling, Shaanxi 712100, China; ^2^Department of Animal & Avian Sciences, University of Maryland, College Park, MD 20742, USA; ^3^Department of Animal Breeding and Genetics, College of Animal Sciences, China Agricultural University, Beijing 100193, China; ^4^Bovine Functional Genomic Laboratory, Animal and Natural Resources Institute, USDA-Agricultural Research Service, Beltsville, MD 20705, USA; ^5^Standerds Division, USDA-Agricultural Marketing Service-National Organic Program, Washington, DC 20250, USA

## Abstract

Beef is one of the leading sources of protein, B vitamins, iron, and zinc in human food. Beef palatability is based on three general criteria: tenderness, juiciness, and flavor, of which tenderness is thought to be the most important factor. In this study, we found that beef tenderness, measured by the Warner-Bratzler shear force (WBSF), was dramatically increased by acute stress. Microarray analysis and qPCR identified a variety of genes that were differentially expressed. Pathway analysis showed that these genes were involved in immune response and regulation of metabolism process as activators or repressors. Further analysis identified that these changes may be related with CpG methylation of several genes. Therefore, the results from this study provide an enhanced understanding of the mechanisms that genetic and epigenetic regulations control meat quality and beef tenderness.

## 1. Introduction

Beef is a source of high-quality nutrition for human populations. Beef palatability is generally determined by three general criteria: tenderness, juiciness, and flavor. Of these factors, beef consumers usually consider tenderness as the most important palatability trait leading to a good eating experience [1–3]. Inconsistency in tenderness has been reported as the most important factor in determining consumer satisfaction with beef quality [4–9]. It is well known that beef tenderness is influenced not only by genetic factors but also environmental aspects. Many studies have been performed on beef quality and tenderness, identifying various important candidate genes [10, 11], quantitative trait loci (QTL), and single-nucleotide polymorphisms (SNPs) [12–20]. High-throughput transcriptomics and proteomics were also used to explore the mechanism of controlling beef quality and tenderness [21–27]. These researches focused much attention on genetic factors influencing beef tenderness. Anecdotally, farmers found that beef produced by cattle which suffered from acute stress, such as injury, surgery, or hardware disease, has much lower quality compared to beef from normal cattle [28–31]. This phenomenon like hardware disease may occur often; therefore the underlying mechanism needs to be explored to better understand what drives beef tenderness and to ultimately improve profitability and efficiency of beef production. So far, we have not seen research which examines the mechanisms of beef quality alteration attributed to acute stress. In this experiment, we found an acute stress event that altered beef tenderness. Since stress is a general phenomenon in beef industry, it is meaningful to explore the biochemical mechanisms on beef quality influenced by acute stress.

In this study, we hypothesized that a one time, acute stress event would alter beef tenderness and quality through gene expression changes, which may be mediated by epigenetic mechanisms. The aims of the research were to further detect the influence of stress on beef tenderness, to explore underlying genes, pathways, and networks regulating beef quality, and to obtain deep insights into the mechanisms of beef tenderness affected by stress.

## 2. Materials and Methods

### 2.1. Sample Preparation and Experimental Design

Seven purebred Angus steers were obtained from the Wye Angus farm (Queenstown, MD, USA). The steers were acclimated to a pelleted forge diet designed to meet maintenance needs. At 10 months of age, 4 steers underwent a surgical procedure that involved anesthetization and placement of a rumen catheter. The surgery was an acute stress event. Three steers received no surgery.

At the approximate age of 1 year, the steers were serially harvested. Immediately after harvest, samples of *longissimus dorsi* (LD) from the right side of the carcass were placed in RNAlater solution (Qiagen, Valencia, CA, USA) at −80°C for further analysis. The carcasses were then chilled for 48 hours at 4°C. Steaks of the LD from the 12~13th rib (2.59 cm) were obtained, vacuum packed, stored at 4°C for a total of 14 days post harvest, and then frozen at −20°C. Once all steaks were obtained, aged, and then frozen, the steaks were thawed at 4°C, cooked to an internal temperature at 70°C, cooled, cored, and then analyzed for WBSF as previously described [32]. All procedures followed the standard animal welfare and used guidelines from the University of Maryland.

### 2.2. RNA Isolation and Microarray Hybridization

 About 20~30 mg LD samples were homogenized in TRizol Reagent (Invitrogen, Carlsbad, CA, USA), and total RNA was extracted as described in the manufacturer's instructions (Invitrogen). Total RNA was purified using DNase I (Qiagen) and the RNA easy Mini column (Qiagen). The RNA was quantified by NanoDrop ND 1000 Spectrophotometer (Thermo Scientific, Wilmington, DE, USA) and RNA integrity determined by 2100 Bioanalyzer (Agilent Technologies, Santa Clara, CA, USA). Agilent 4 × 44 K bovine microarrays were used in this study. This array was designed based on the whole bovine genome sequence. RNAs from all samples were mixed to an RNA pool as a reference sample. Two microgram RNA of each sample was labeled with Cy3 using the Agilent Quick-Amp labeling Kit (Agilent Technologies) while 2 *μ*g reference RNA was labeled with Cy5. Then 825 ng of the appropriate Cy3- and Cy5-labeled complementary RNAs (cRNA) were hybridized to the 4 × 44 K Agilent bovine arrays, and a total of 7 arrays were hybridized.

### 2.3. Data Collection, Normalization, and Analysis

 Following stringency washes, slides were scanned on an Agilent G2505B microarray scanner, and the resulting image files were analyzed with Agilent Feature Extraction software (Version 9.5.1). All procedures were carried out according to the manufacturer's protocols. Background adjustment, quantile normalization across 7 microarrays, and statistical analysis were performed using the Limma package (linear models for microarray data). Significantly expressed probes, in the comparisons of stress *versus *nonstress, were screened for subsequent pathway and network analysis.

### 2.4. Clustering and Network Analysis

 Hierarchical clustering of expression profiles was performed using Cluster 3.0 [33]. The data were further normalized. Average linkage clustering was performed and visualized using Treeview [33]. The initial information on gene ontology (GO) functions and functional relevance of significantly expressed probes were obtained from the Gene Ontology Enrichment Analysis Software Toolkit (GOEAST) [34]. Ingenuity pathway analysis (IPA) (Ingenuity System, http://www.ingenuity.com/) was used to generate networks and assess statistically relevant biofunctions and canonical pathways. A dataset containing gene name, logFC (fold change), and *P* value was uploaded and mapped to corresponding expression genes in the Ingenuity knowledge database. The biofunctional analysis identified “molecular and cellular function” and “physiological system development and function.” Canonical pathway analysis identified pathways most significantly represented in the dataset. The significance between the dataset and the canonical pathway was measured using Fisher's exact test for a *P* value and a Benjamini-Hochberg correction for multiple testing applied.

### 2.5. Quantitative Real-Time PCR

 To validate the microarray results, genes were selected based on their functions and significance in the results of microarray. Quantitative real-time PCR primers were designed online with primer 3 (http://frodo.wi.mit.edu/primer3/). The uniqueness of the designed primer pairs was validated by a BLAST homology search (http://www.ncbi.nlm.nih.gov/blast/Blast.cgi) to ensure that homologous genes were not cross-amplified by the same primer pair. Whenever possible, primers were designed to span intron/exon boundaries. All primers for target genes examined are given in Supplementary Table 1 (see Supplementary Materials available online at doi:10.1155/2012/756284).

Total RNA from the same LD sample was isolated, purified, quantified, and ingenuity determined in the same procedure as the microarray experiment. The first strand cDNA was synthesized from 1 *μ*g of total RNA using SuperScript II Reverse Transcriptase (Invitrogen) with oligo(dT) primers (Invitrogen). Samples were then analyzed by real-time PCR using an iCycler iQ PCR system (Bio-Rad, Hercules, CA, USA). The real-time PCR reactions were performed in a final volume of 20 *μ*L with a QuantiTect SYBR Green PCR Kit (Qiagen) according to the manufacturer's instruction. The efficiencies of target genes and *glyceraldehyde-3-phosphate dehydrogenase* (*GAPDH*) amplification were investigated by performing a serial dilution of total RNA (1 *μ*g to 0.1 ng) following recommendations [35]. The mRNA expression was normalized against the housekeeping gene *GAPDH*, the most commonly used housekeeping gene [36]. Each real-time PCR program was run for 15 minutes at 95°C, followed by 40 repeats of 15 s at 94°C, 30 s at 58°C, and 30 s at 70°C. Data were analyzed using the 2^−∆∆CT^ method [35]. The statistical significance of the raw Ct values representing differences in mRNA expression level was determined by two-tailed student's *t*-test. The correlation analysis between real-time PCR and microarray expression data was conducted using CORR procedure of SAS (SAS Inst. Inc.) [37].

### 2.6. Methylation Pattern Analysis of the Significant Genes

Genes were selected based on their functions and significance in microarray and CpG island enrichment on their promoters. CpG island distributions in promoter regions were checked using the UCSC Genome browser (http://genome.ucsc.edu/), and promoter sequences of these genes were downloaded from NCBI (http://www.ncbi.nlm.nih.gov/). After CGs were replaced to YGs and then Cs were converted to Ts, the sequence was input into the PSQ Assay Design software (PyroMark ID, Qiagen), and bisulfited-PCR primers were designed to amplify these promoter regions. The primers for methylation detection, including forward primer, reverse primer, and sequencing, are listed in the Supplementary Table 2.

Genomic DNA was isolated from the same LD sample using NucleoSpin kit (Macherey-Nagel, Bethlehem, PA, USA). One microgram of DNA was treated with a sodium bisulfate conversion reagent (EZ DNA Methylation Golden Kit) (Zymo Research Corporation, Irvine, CA, USA) according to the instruction manual. The amplification efficiencies of primers were investigated by performing dilution series of bisulfited DNA and PCR. Then the diluted bisulfited DNA served as the template for bisulfited PCR using the HotStar Taq polymerase (Qiagen), and a biotin-labeled universal primer was added in each PCR reaction. Pyrosequencing analysis by Pyro Q-CpG system (PyroMark ID, Qiagen) was performed to detect methylation level of each CpG site using 30 *μ*L of PCR products, which were analyzed by gel electrophoresis to confirm that the PCR amplified successfully [38].

## 3. Results

### 3.1. Tenderness of Angus Beef in This Experiment

**Figure 1:**
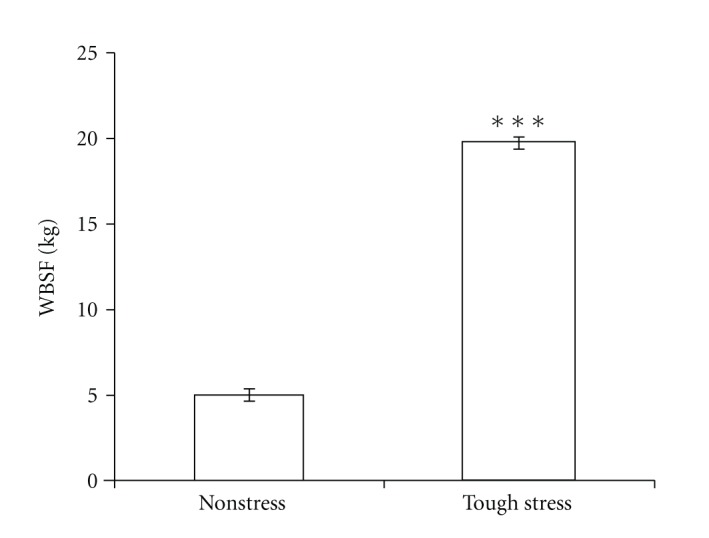
The results of WBSF between nonstress group and tough-stress group. Data are shown in mean ± SE (***P < 0.0001).

Four steers were anesthetized and given a surgery to place a rumen catheter. This surgical procedure is an acute stress event compared with 3 controls. To evaluate variation of beef tenderness caused by this acute stress, the Warner-Bratzler shear forces (WBSF) were measured [39]. The WBSF results showed that the stress group was much tougher than the control (nonstress) group (*P* < 0.0001) (Figure 1). In addition, all of the carcasses were qualitatively graded. Thus differences in marbling did not contribute to any differences in tenderness. Further, all of the steers were approximately 1 year of age, so age effects due to collagen crosslinking should be minimized. Then, we performed further microarray analysis based on these stress and control groups.

### 3.2. Differentially Expressed Genes in Divergent Stress Status

**Figure 2:**
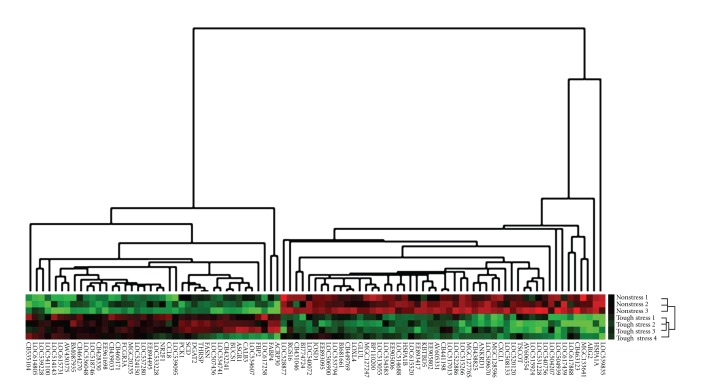
Cluster analysis of significant genes in microarray. These genes were visualized with Treeview after hierarchical clustering. Each gene is represented by a single row of colored boxes; each individual from two groups is represented by a single column. Red color indicates upregulated while green indicates downregulated. Genes that were expressed at higher levels are assigned progressively brighter shades of red while genes expressed at lower levels are assigned progressively brighter shades of green.

 To determine the differentially expressed genes between these two groups of differential stress status, cDNA microarray analysis was done using LD samples. With the aid of the Limma package in Bioconductor, we selected significant expressed probes based on a stringent statistical significance threshold (*P* < 0.05, |*lgFC*| > 1.5, and false discovery rate (FDR) <0.3). The results showed that a total of 215 probes were significantly differentially expressed, which attribute 137 unique probes. Of these 137 probes, 102 were assigned to genes while 35 were assigned to ESTs. Among these 137 genes (or ESTs), 73 were downregulated while 64 were upregulated in tough stress compared to nonstress. To reveal the overall expression profile of these significant genes (or ESTs) in these two groups, clustering analysis was performed as previously described [33]. The visualization showed that the expression pattern of these significant genes was apparently different between these two groups. Also, most of the genes had highly consistently expression level within each group (Figure 2).

### 3.3. Quantitative Real-Time PCR Results

**Figure 3:**
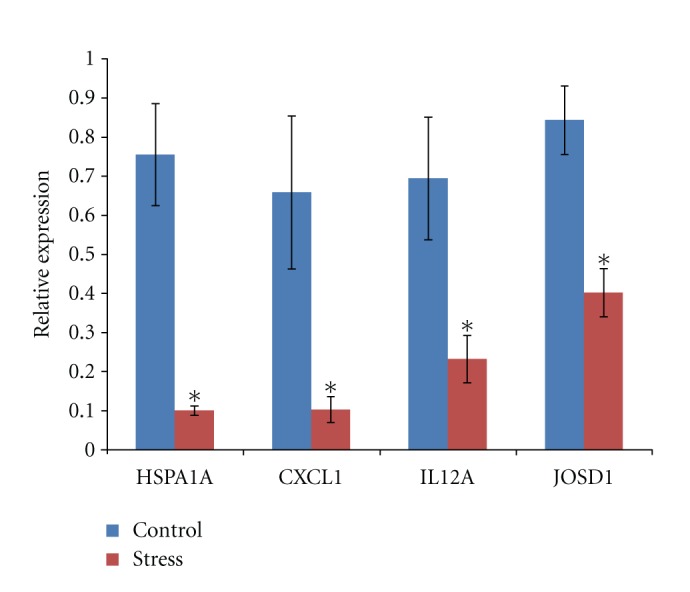
The q-RT-PCR results showed the relative expression of 4 genes. All the 4 genes were significantly differentially expressed between stress group and control group (*P < 0.05).

 Four genes, *heat shock *70 kDa* protein 1A (HSPA1A), chemokine (C-X-C motif) ligand 2 (CXCL1), interleukin 12A (IL12A), *and* Josephin domain containing 1 (JOSD1)* which function in immune response and also significantly differentially expressed in microarray between tough stress and nonstress, were chosen to perform RT-PCR to validate microarray results. qRT-PCR results showed that gene expression patterns of these 4 genes were of significant difference between these two groups (Figure 3) (*P* < 0.05). In addition, the dysregulation directions and fold changes of these genes were highly consistent between RT-PCR and microarray (*R* ^2^ = 0.9595).

### 3.4. Functional Annotation of Significantly Differentially Expressed Genes

To investigate the functionality that these significantly expressed genes are involved in, GO term analysis was employed and the results showed that significantly differentially expressed genes in GO biological process terms were enriched in regulation of fatty acid and protein metabolic process, receptor biosynthetic process, receptor of myeloid cell apoptosis, negative regulation of neuron differentiation, response to glucose stimulus, monocarboxylic acid metabolic process, gluconeogenesis, and so forth. In cellular component category, GO terms related to extracellular region part and extracellular space. The molecular function category of GO term showed that cytokine activity, cytokine receptor binding, and IgG binding were enriched. Summaries of the enriched GO term categories for significantly differentially expressed genes are shown in Table 1.

**Figure 4:**
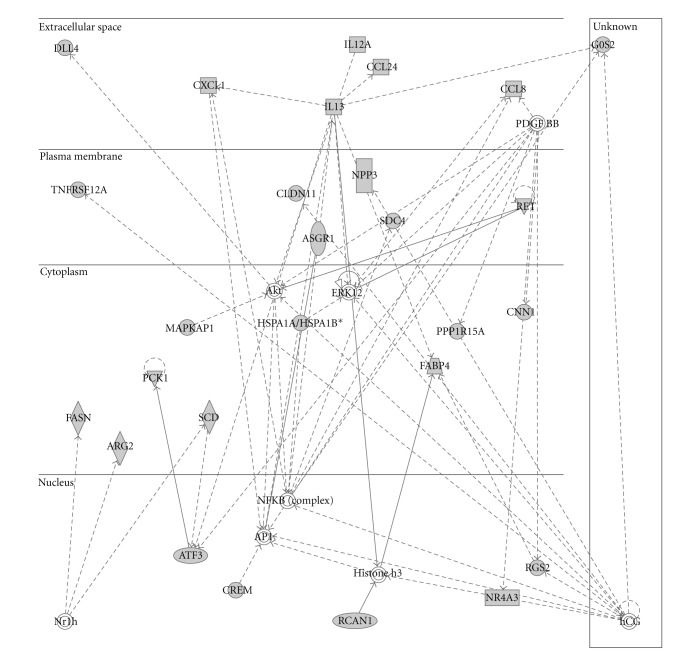
The top No. 1 network significantly differentially expressed genes involved. Solid line represents direct interaction and dash line represents indirect interaction.

To further visualize the pathways and networks these significantly differentially expressed genes functioned in, IPA was conducted. After uploading the gene set, 79 from 102 genes mapped to the IPA knowledge database. Analysis results showed that carbohydrate metabolism, gene expression, lipid metabolism, small-molecule biochemistry, and molecular transport were ranked in the top 5 of “molecular and cellular functions.” While differential regulation of cytokine production in macrophages and T helper cells by *IL-17A* and *IL-17F*, differential regulation of cytokine in intestinal epithelial by *IL-17A* and *IL-17F*, LXR/RXR activation, TR/RXR activation, and thyroid cancer signaling were among the top canonical pathways. The most significant networks functioned in cellular growth and proliferation, cellular movement, and lipid metabolism. Summaries of the enriched networks, their functions are shown in Table 2, and graphical networks are represented in Figure 4, Supplementary Figure 1, and Supplementary Figure 2.

### 3.5. Methylation Patterns Analysis of Significantly Differentially Expressed Genes

**Figure 5:**
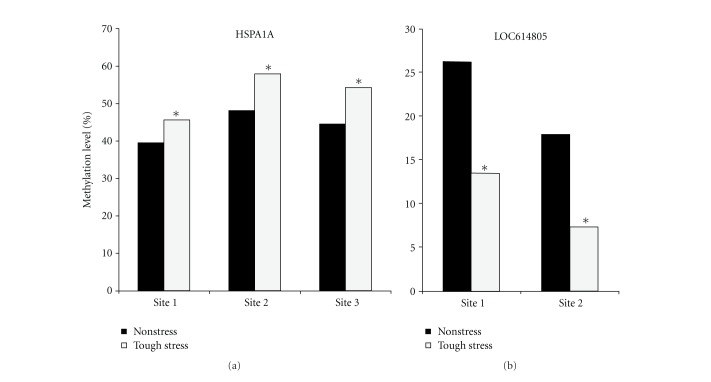
Significant changes of methylation level in the promoter region of significant genes between tough-stress and nonstress groups. *x*-axis represents CpG sites on the promoter; *y*-axis represents DNA methylation level of these sites (*P < 0.05).

To ascertain whether a stress stimulus induces any epigenetic alterations, DNA methylation patterns of several genes were checked. We blasted these significant genes in the bovine genome; 6 differentially expressed genes enriched with CpG islands in promoter regions were selected to detect methylation levels in LD muscle using pyrosequencing. The results showed that the methylation levels of 3 CpG sites in the promoter of *HSPA1A* significantly increased in the stress group compared with the control group (*P* < 0.05) and the methylation levels of 2 CpG sites in *LOC614805* significantly decreased in the stress group compared with the control group (*P* < 0.05) (Figure 5). Combining the methylation patterns and gene expression levels in microarray, we found that for these genes the methylation levels increased while the gene expression level decreased and *vice versa*, implying that the gene expression levels were inversely correlated with the methylation levels in promoter regions in this study.

## 4. Discussion

Beef tenderness is deemed the most important palatability attribute. Thus, improving tenderness and providing consistently tender beef are the priority for beef industry. More efforts have been put on factors influencing production, including breed, sex, feed, handling, environment, finishing weight and age at slaughter, and so forth [40]. In this study, after a one-time acute stress event, those cattle produced beef with significantly higher WBSF, indicating that acute stress has tremendous influence on beef tenderness. From cDNA microarray analysis in LD muscles of control and stress groups, we identified 137 differently expressed genes (or ESTs) related to variations on stress status and beef quality.

Notably, acute stress can induce strong immune response. The pathway analysis on significantly differentially expressed genes showed that several chemokine or cytokine encoded genes, such as *interleukin 12A (IL12A), interleukin 13 (IL13), chemokine (C-C motif) ligand 8 (CCL8), chemokine (C-C motif) ligand 24 (CCL24), *and *chemokine (C-X-C motif) ligand 1 (CXCL1),* were involved in immune response, implicating that immune response to this acute stress by chemokine or cytokine may play important roles in beef tenderness variation. Chemokines play fundamental roles in the development, homeostasis, and function in the immune system, especially in skeletal muscle regeneration, although the mechanisms involved are still poorly elucidated [41–44]. Some chemokines, such as *CXCL1*, can directly stimulate myoblast migration and are involved in the cellular differentiation process [45]. Meanwhile, cytokines are proteinaceous signaling compounds that are major mediators of the immune response. Multiple findings indicate that cytokines influence different physiologic functions of skeletal muscle cells, such as anabolic and catabolic processes and programmed cell death [46]. It has been found that cytokines can regulate different stages of myocyte development, including proliferation and differentiation of myoblasts, expression of myogenic proteins, and fusion of myotubes [47]. Some studies also found that cytokines were important regulatory molecules in the complex network of signals that control muscle protein breakdown [46]. Here, these chemokine- or cytokine-encoded genes were dysregulated in stress group, implying that a complex network, combining these chemokine and cytokine regulators together, may coregulate muscle protein development and breakdown and even postmortem proteolysis during carcass ageing, which directly influences meat quality and tenderness. But the mechanism needs to be further explored in the future research.

The acute stress is associated with regulation of metabolic process. The most significant GO terms identified were enriched with genes involved in regulation of metabolism, including *activating transcription factor 3 (ATF3),* r*egulator of Calcineurin 1 (RCAN1), adiponectin, C1Q and collagen domain containing (ADIPOQ), growth arrest and DNA-damage-inducible, gamma (GADD45G), single-minded homolog 1 (SIM1), nuclear receptor subfamily 2, group F, member 1 (NR2F1), zinc fingers and homeoboxes 3 (ZHX3), GS homeobox 2 (Gsx2), *and so forth. Further study of the functions of these genes determined that they played roles in regulation of metabolism as activators or repressors. ATF3 is a member of the mammalian activation transcription factor. This protein binds the cAMP response element (CRE) and represses transcription from promoters with ATF sites [48]. *RCAN1* is a dose-sensitive gene whose overexpression has been linked to disease neuropathology and to the response of cells to stress stimuli. The protein encoded by this gene interacts with calcineurin A and inhibits calcineurin-dependent signaling pathways [49, 50]. *ADIPOQ* is involved in the control of fat metabolism and insulin sensitivity [51]. This protein can stimulate AMPK phosphorylation and activation in the liver and the skeletal muscle, enhancing glucose utilization and fatty acid combustion [52]. SIM1 is a basic Helix-Loop-Helix/Per-Arnt-SIM (bHLH-PAS) transcription factor [53]. It is reported that *SIM1* expression is associated with the early step of muscle progenitor cell migration in chick and mouse [54]. Two SNPs on this gene were found to be associated with carcass and meat quality traits in a porcine population [55]. *GADD45G* is involved in stress signaling in response to physiological or environmental stressors, and this protein functions as stress sensors [56]. Protein NR2F1 consists of ligand-inducible transcription factors and can stimulate initiation of transcription [57]. *ZHX3* encodes a member of the zinc fingers and homeoboxes (ZHX) gene family. In the nucleus, the dimerized ZHX3 protein interacts with the a subunit of the ubiquitous transcription factor and may function as a transcriptional repressor [58]. Gsx2 can regulate the balance between proliferation and differentiation of the neuronal progenitor [59, 60]. Taking together, all of these genes encoding activators or repressors dysregulated between stress and control groups, but how they cooperate together to regulate muscle proliferation and differentiation and beef tenderness needs to be further explored.

Most importantly, we found that in some genes the methylation levels increased while expression levels decreased and *vice versa*, implicating that the gene expression levels were inversely correlated with the methylation levels in promoter regions, which supports the previous report that DNA methylation represses gene expression [38]. Also, detection of different DNA methylation patterns of these several genes further supports our hypothesis that epigenetic mechanisms involve in the acute stimulus through altering expression of genes, suggesting that epigenetic mechanisms may at least partially determine the beef tenderness in this study.

Of these methylated while dysregulated genes, *HSPA1A* has been identified to be related with beef tenderness. The heat shock proteins, encoded by this gene family, are primarily intracellular molecular chaperones involved in cell survival and in protecting the cell from a stressful condition and exert profound effects on the host's response to autoimmunity and unknown stressors [61–63]. This study identified epigenetic regulation of heat shock proteins in response to acute stress. With epigenetic regulation, stress events very early in life could persist and could be a factor in tough beef from cattle that are healthy and not under stress at the time of slaughter. Thus, to further elucidate the mechanism of acute stress, such as hardware disease, in determining beef tenderness, a comprehensive analysis between genome-wide DNA methylation and this microarray results will be further investigated, which will help us explore the genetic and epigenetic factors coregulating gene expression and cooperatively influencing beef tenderness.

In summary, acute stress had a significant influence on beef tenderness. The differentially expressed genes were involved in immune response and genes encoding activators or repressors, suggesting that external stresses play important roles in tenderness variation. Further analysis found that DNA methylation was also associated with beef quality. Future research will explore the mechanisms how genetic and epigenetic factors determine meat quality and beef tenderness.

## Supplementary Material

Supplementary Figure 1: The top 2 # network significantly differentially expressed genes involved in. Solid line represents direct interaction and dash line represents indirect interaction.
Supplementary Figure 2: The top 3 # network significantly differentially expressed genes involved in. Solid line represents direct interaction and dash line represents indirect interaction.
Supplementary Table 1: Primers for RT-PCR.
Supplementary Table 2: Primers for bisulfited-PCR.

## Figures and Tables

**Table 1:** Significant GO terms 137 significant probes were involved in.

GO ID	Ontology	Term	Gene number	*P* value
GO: 0019222	biological_process	Regulation of metabolic process	16	0.032133
GO: 0031323	biological_process	Regulation of cellular metabolic process	15	0.032133
GO: 0032502	biological_process	Developmental process	15	0.034002
GO: 0030154	biological_process	Cell differentiation	12	0.018268
GO: 0048869	biological_process	Cellular developmental process	12	0.021079
GO: 0048523	biological_process	Negative regulation of cellular process	10	0.043805
GO: 0042221	biological_process	Response to chemical stimulus	9	0.042394
GO: 0006955	biological_process	Immune response	7	0.032133
GO: 0032787	biological_process	Monocarboxylic acid metabolic process	6	0.021079
GO: 0006006	biological_process	Glucose metabolic process	5	0.016802
GO: 0019318	biological_process	Hexose metabolic process	5	0.023776
GO: 0005996	biological_process	Monosaccharide metabolic process	5	0.031139
GO: 0050873	biological_process	Brown fat cell differentiation	4	0.006619
GO: 0045444	biological_process	Fat cell differentiation	4	0.016802
GO: 0006094	biological_process	Gluconeogenesis	3	0.023776
GO: 0019319	biological_process	Hexose biosynthetic process	3	0.025047
GO: 0046364	biological_process	Monosaccharide biosynthetic process	3	0.032133
GO: 0032370	biological_process	Positive regulation of lipid transport	2	0.046664
GO: 0045598	biological_process	Regulation of fat cell differentiation	2	0.046664
GO: 0010871	biological_process	Negative regulation of receptor Biosynthetic process	2	0.046664
				
GO: 0044421	cellular_component	Extracellular region part	9	0.032203
GO: 0005615	cellular_component	Extracellular space	8	0.023776
				
GO: 0005125	molecular_function	Cytokine activity	5	0.024213
GO: 0005126	molecular_function	Cytokine receptor binding	5	0.032133
GO: 0019864	molecular_function	IgG binding	2	0.046664

**Table 2:** Top significant pathways and functions.

ID	Molecules in network	Score	Focus molecules	Top functions
1	Akt, Ap1, ARG2, ASGR1, ATF3, CCL8, CCL24, CLDN11, CNN1, CREM, CXCL1, DLL4, ERK1/2, FABP4, FASN, G0S2, hCG, Histone h3, HSPA1A/HSPA1B, IL13, IL12A, MAPKAP1, NFkB (complex), NPR3, Nr1h, NR4A3, PCK1, PDGF, PPP1R15A, RCAN1, RET, RGS2, SCD, SDC4, TNFRSF12A	57	27	Cellular growth and proliferation, cellular movement, lipid metabolism

2	Aconitase, ANKRD1, AP2B1, APCS, ASPM, BLNK, CA12, CDX2, DGAT2, EFNA3, ENPP1, FCGR3A, GADD45G, HAS1, HDGF, HNF1A, HTT, IFRD1, IgG, IL1B, ITPA, KIF20A, NGEF, PFK, PPL, RARA, RNA polymerase II, S100G, SERPING1, SGK2, SIM1, SOAT2, TGFB1, UGT1A8, YY1	21	13	Embryonic development, reproductive system development and function, nutritional disease

3	ABLIM, ACP2, ACSL5, ADCY9, ARPC1A, ATPIF1, CIDEC, CREBBP, DUSP3, DYRK3, FBP2, FSH, GLUL, GNLY, HGD, HOXB6, ING2, LEP, Lh, LHCGR, LOC81691, NR2F1, NR3C1, NR5A1, P4HA2, PDLIM3, PLCL1, POP5, PRKX, RAB5C, RGS5, RGS16, SREBF1, THRSP, TLK1	11	8	Drug metabolism, endocrine system development and function, lipid metabolism

4	NAALAD2, RORA	2	1	Amino acid metabolism, small molecule biochemistry, gene expression

5	FIGLA, TEKT1	2	1	Cell cycle, reproductive system development and function, cell death

6	ZHX1, ZHX3	2	1	Gene expression

7	AGTR1, HSPA6, RFWD2	2	1	Cardiovascular system development and function, organ morphology, organismal injury and abnormalities

8	AHR, GPI, STEAP4	2	1	Cell cycle, cell morphology, cell-to-cell signaling and interaction

9	CBX4, HOXA2, MBD1, MEOX1	2	1	Gene expression, embryonic development, organismal development

10	FBL, NFE2, PSMD14, STMN1, TUBB1	2	1	Cell morphology, hematological system development and function, inflammatory response

11	DMD, SGCA, SGCB, SGCD, SGCE, SGCG	1	1	Developmental disorder, genetic disorder, skeletal and muscular disorders

## References

[1] Robinson DL, Ferguson DM, Oddy VH, Perry D, Thompson J (2001). Genetic and environmental influences on beef tenderness. *Australian Journal of Experimental Agriculture*.

[2] Watson R, Gee A, Polkinghorne R, Porter M (2008). Consumer assessment of eating quality—development of protocols for Meat Standards Australia (MSA) testing. *Australian Journal of Experimental Agriculture*.

[3] Huffman KL, Miller MF, Hoover LC, Wu CK, Brittin HC, Ramsey CB (1996). Effect of beef tenderness on consumer satisfaction with steaks consumed in the home and restaurant. *Journal of Animal Science*.

[4] Boleman SJ, Boleman SL, Miller RK (1997). Consumer evaluation of beef of known categories of tenderness. *Journal of Animal Science*.

[5] Brady DE (1937). A study of the factors influencing tenderness and texture of beef. *Journal of Animal Science*.

[6] Goodson KJ, Morgan WW, Reagan JO (2002). Beef Customer Satisfaction: factors affecting consumer evaluations of clod steaks. *Journal of Animal Science*.

[7] Jeremiah LE (1992). A review of factors influencing consumption, selection and acceptability of meat purchases. *Journal of Consumer Studies & Home Economics*.

[8] Kim R (2003). Factors influencing consumers’ decision to purchase beef: a South Korean case study. *Journal of International Food and Agribusiness Marketing*.

[9] Behrends JM, Goodson KJ, Koohmaraie M (2005). Beef customer satisfaction: USDA quality grade and marination effects on consumer evaluations of top round steaks. *Journal of Animal Science*.

[10] Lebret B, Le Roy P, Monin G (1999). Influence of the three RN genotypes on chemical composition, enzyme activities, and myofiber characteristics of porcine skeletal muscle. *Journal of Animal Science*.

[11] Di Stasio L, Sartore S, Albera A (2002). Lack of association of GH1 and POU1F1 gene variants with meat production traits in Piemontese cattle. *Animal Genetics*.

[12] Barendse W, Harrison BE, Bunch RJ, Thomas MB (2008). Variation at the Calpain 3 gene is associated with meat tenderness in zebu and composite breeds of cattle. *BMC Genetics*.

[13] Hocquette JF, Renard G, Levéziel H, Picard B, Cassar-Malek I (2006). The potential benefits of genetics and genomics to improve beef quality—a review. *Animal Science Papers and Reports*.

[14] Gill JL, Bishop SC, McCorquodale C, Williams JL, Wiener P (2009). Association of selected SNP with carcass and taste panel assessed meat quality traits in a commercial population of Aberdeen Angus-sired beef cattle. *Genetics Selection Evolution*.

[15] Chen FY, Niu H, Wang JQ (2011). Polymorphism of DLK1 and CLPG gene and their association with phenotypic traits in Chinese cattle. *Molecular Biology Reports*.

[16] Iglesias PP, Caffaro ME, Amadio AF, Arias Mañotti A, Poli MA (2010). CAPN1 markers in three Argentinean cattle breeds: report of a new InDel polymorphism within intron 17. *Molecular Biology Reports*.

[17] Iwanowska A, Grześ B, Mikołajczak B (2011). Impact of polymorphism of the regulatory subunit of the *μ*-calpain (CAPN1S) on the proteolysis process and meat tenderness of young cattle. *Molecular Biology Reports*.

[18] Fan YY, Zan LS, Fu CZ (2010). Three novel SNPs in the coding region of PPAR*γ* gene and their associations with meat quality traits in cattle. *Molecular Biology Reports*.

[19] Davis GP, Moore SS, Drinkwater RD (2008). QTL for meat tenderness in the M. longissimus lumborum of cattle. *Animal Genetics*.

[20] Gao Y, Zhang R, Hu X, Li N (2007). Application of genomic technologies to the improvement of meat quality of farm animals. *Meat Science*.

[21] Morzel M, Terlouw C, Chambon C, Micol D, Picard B (2008). Muscle proteome and meat eating qualities of Longissimus thoracis of “Blonde d’Aquitaine” young bulls: a central role of HSP27 isoforms. *Meat Science*.

[22] Mullen AM, Stapleton PC, Corcoran D, Hamill RM, White A (2006). Understanding meat quality through the application of genomic and proteomic approaches. *Meat Science*.

[23] Sawdy JC, Kaiser SA, St-Pierre NR, Wick MP (2004). Myofibrillar 1-D fingerprints and myosin heavy chain MS analyses of beef loin at 36 h postmortem correlate with tenderness at 7 days. *Meat Science*.

[24] Bernard C, Cassar-Malek I, Le Cunff M, Dubroeucq H, Renand G, Hocquette JF (2007). New indicators of beef sensory quality revealed by expression of specific genes. *Journal of Agricultural and Food Chemistry*.

[25] Zhang Y, Zan L, Wang H (2010). Screening candidate genes related to tenderness trait in Qinchuan cattle by genome array. *Molecular Biology Reports*.

[26] Zapata I, Zerby HN, Wick M (2009). Functional proteomic analysis predicts beef tenderness and the tenderness differential. *Journal of Agricultural and Food Chemistry*.

[27] Koohmaraie M, Kent MP, Shackelford SD, Veiseth E, Wheeler TL (2002). Meat tenderness and muscle growth: is there any relationship?. *Meat Science*.

[28] King DA, Schuehle Pfeiffer CE, Randel RD (2006). Influence of animal temperament and stress responsiveness on the carcass quality and beef tenderness of feedlot cattle. *Meat Science*.

[29] Grandin T (1980). The effect of stress on livestock and meat quality prior to and during slaughter [Cattle, pigs and sheep]. *International Journal for the Study of Animal Problems*.

[30] Warriss PD (1990). The handling of cattle pre-slaughter and its effects on carcass and meat quality. *Applied Animal Behaviour Science*.

[31] Remignon H, Mills AD, Guemene D (1998). Meat quality traits and muscle characteristics in high or low fear lines of Japanese quails (Coturnix japonica) subjected to acute stress. *British Poultry Science*.

[32] Zhao C, Tian F, Yu Y (2012). Muscle transcriptomic analyses in Angus cattle with divergent tenderness. *Molecular Biology Reports*.

[33] Eisen MB, Spellman PT, Brown PO, Botstein D (1998). Cluster analysis and display of genome-wide expression patterns. *Proceedings of the National Academy of Sciences of the United States of America*.

[34] Zheng Q, Wang XJ (2008). GOEAST: a web-based software toolkit for Gene Ontology enrichment analysis. *Nucleic Acids Research*.

[35] Livak KJ, Schmittgen TD (2001). Analysis of relative gene expression data using real-time quantitative PCR and the 2^−ΔΔ*C*_T_^ method. *Methods*.

[36] Robinson TL, Sutherland IA, Sutherland J (2007). Validation of candidate bovine reference genes for use with real-time PCR. *Veterinary Immunology and Immunopathology*.

[37] Wang YH, Bower NI, Reverter A (2009). Gene expression patterns during intramuscular fat development in cattle. *Journal of Animal Science*.

[38] Yu Y, Zhang H, Tian F (2008). Quantitative evaluation of DNA methylation patterns for ALVE and TVB genes in a neoplastic disease susceptible and resistant chicken model. *PLoS One*.

[39] Rodas-González A, Huerta-Leidenz N, Jerez-Timaure N, Miller MF (2009). Establishing tenderness thresholds of Venezuelan beef steaks using consumer and trained sensory panels. *Meat Science*.

[40] Fervers B, Burgers JS, Voellinger R (2011). Modern approaches to enhancing beef quality. *Tehnologija Mesa*.

[41] Contreras-Shannon V, Ochoa O, Reyes-Reyna SM (2007). Fat accumulation with altered inflammation and regeneration in skeletal muscle of CCR2−/− mice following ischemic injury. *American Journal of Physiology*.

[42] Keylock KT, Vieira VJ, Wallig MA, DiPietro LA, Schrementi M, Woods JA (2008). Exercise accelerates cutaneous wound healing and decreases wound inflammation in aged mice. *American Journal of Physiology*.

[43] Ochoa O, Sun D, Reyes-Reyna SM (2007). Delayed angiogenesis and VEGF production in CCR2−/− mice during impaired skeletal muscle regeneration. *American Journal of Physiology*.

[44] Warren GL, O’Farrell L, Summan M (2004). Role of CC chemokines in skeletal muscle functional restoration after injury. *American Journal of Physiology*.

[45] Nedachi T, Hatakeyama H, Kono T, Sato M, Kanzaki M (2009). Characterization of contraction-inducible CXC chemokines and their roles in C_2_C_12_ myocytes. *American Journal of Physiology*.

[46] Zoico E, Roubenoff R (2002). The role of cytokines in regulating protein metabolism and muscle function. *Nutrition Reviews*.

[47] Gadient RA, Patterson PH (1999). Leukemia inhibitory factor, interleukin 6, and other cytokines using the GP130 transducing receptor: roles in inflammation and injury. *Stem Cells*.

[48] Pan YX, Chen H, Thiaville MM, Kilberg MS (2007). Activation of the ATF3 gene through a co-ordinated amino acid-sensing response programme that controls transcriptional regulation of responsive genes following amino acid limitation. *Biochemical Journal*.

[49] Sun X, Wu Y, Chen B (2011). Regulator of calcineurin 1 (RCAN1) facilitates neuronal apoptosis through caspase-3 activation. *The Journal of Biological Chemistry*.

[50] Liu H, Wang P, Song W, Sun X (2009). Degradation of regulator of calcineurin 1 (RCAN1) is mediated by both chaperone-mediated autophagy and ubiquitin proteasome pathways. *The FASEB Journal*.

[51] Kondo H, Shimomura L, Matsukawa Y (2002). Association of adiponectin mutation with type 2 diabetes: a candidate gene for the insulin resistance syndrome. *Diabetes*.

[52] Yamauchi T, Kadowaki T (2008). Physiological and pathophysiological roles of adiponectin and adiponectin receptors in the integrated regulation of metabolic and cardiovascular diseases. *International Journal of Obesity*.

[53] Michaud JL, Rosenquist T, May NR, Fan CM (1998). Development of neuroendocrine lineages requires the bHLH-PAS transcription factor SIM1. *Genes and Development*.

[54] Coumailleau P, Duprez D (2009). Sim1 and Sim2 expression during chick and mouse limb development. *International Journal of Developmental Biology*.

[55] Chen Z, Zhao X, Hao Z, Jiang X, Guo X, Xu N (2011). Molecular characteristics of porcine SIM1 gene and its variants association with carcass and meat quality traits. *Journal of Animal and Veterinary Advances*.

[56] Liebermann DA, Hoffman B (2008). Gadd45 in stress signaling. *Journal of Molecular Signaling*.

[57] Brown KK, Alkuraya FS, Matos M, Robertson RL, Kimonis VE, Morton CC (2009). NR2F1 deletion in a patient with a de novo paracentric inversion, inv(5)(q15q33.2), and syndromic deafness. *American Journal of Medical Genetics, Part A*.

[58] Yamada K, Kawata H, Shou Z (2003). Analysis of zinc-fingers and homeoboxes (ZHX)-1-interacting proteins: molecular cloning and characterization of a member of the ZHX family, ZHX3. *Biochemical Journal*.

[59] Illes JC, Winterbottom E, Isaacs HV (2009). Cloning and expression analysis of the anterior ParaHox genes, *Gsh1* and *Gsh2* from Xenopus tropicalis. *Developmental Dynamics*.

[60] Waclaw RR, Wang B, Pei Z, Ehrman LA, Campbell K (2009). Distinct temporal requirements for the homeobox gene *Gsx2* in specifying striatal and olfactory bulb neuronal fates. *Neuron*.

[61] Izawa S, Kita T, Ikeda K, Inoue Y (2008). Heat shock and ethanol stress provoke distinctly different responses in 3′-processing and nuclear export of HSP mRNA in Saccharomyces cerevisiae. *Biochemical Journal*.

[62] Remakova M, Skoda M, Faustova M (2011). The expression regulation of the HSPA1B gene in patients with myositis is not dependent on the presence of HLA-DRB1* 03 risk allele. *Annals of the Rheumatic Diseases*.

[63] Kaur P, Hurwitz MD, Krishnan S, Asea A (2011). Combined hyperthermia and radiotherapy for the treatment of cancer. *Cancers*.

