# Supplementary material for: Functional Genomic Analysis of Variation on Beef Tenderness Induced by Acute Stress in Angus Cattle
Source: Comp Funct Genomics. 2012 Apr 12;2012:756284. doi: 10.1155/2012/756284 (PMC3332163; doi:10.1155/2012/756284)
Supplement: Supplementary file 1 — Supplementary Figure 1: The top 2 # network significantly differentially expressed genes involved in. Solid line represents direct interaction and dash line represents indirect interaction. Supplementary Figure 2: The top 3 # network significantly differentially expressed genes involved in. Solid line represents direct interaction and dash line represents indirect interaction. Supplementary Table 1: Primers for RT-PCR. Supplementary Table 2: Primers for bisulfited-PCR. [file 756284.f1.pdf]

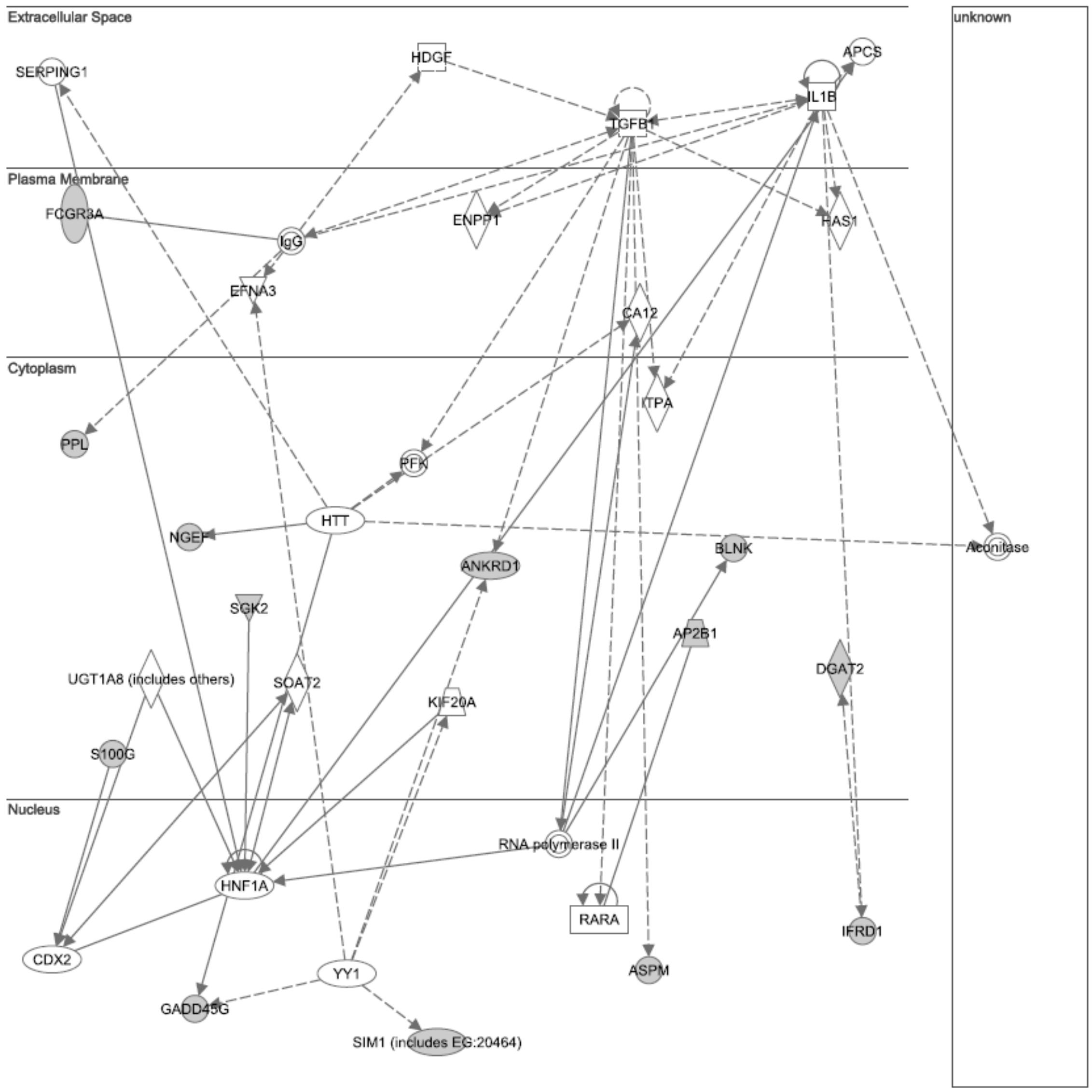

Supplementary Fig. 1 The top 2 # network significantly differentially expressed genes involved in. Solid line represents direct interaction and dash line represents indirect interaction.
