# Supplementary material for: Functional Genomic Analysis of Variation on Beef Tenderness Induced by Acute Stress in Angus Cattle
Source: Comp Funct Genomics. 2012 Apr 12;2012:756284. doi: 10.1155/2012/756284 (PMC3332163; doi:10.1155/2012/756284)
Supplement: Supplementary file 2 [file 756284.f2.pdf]

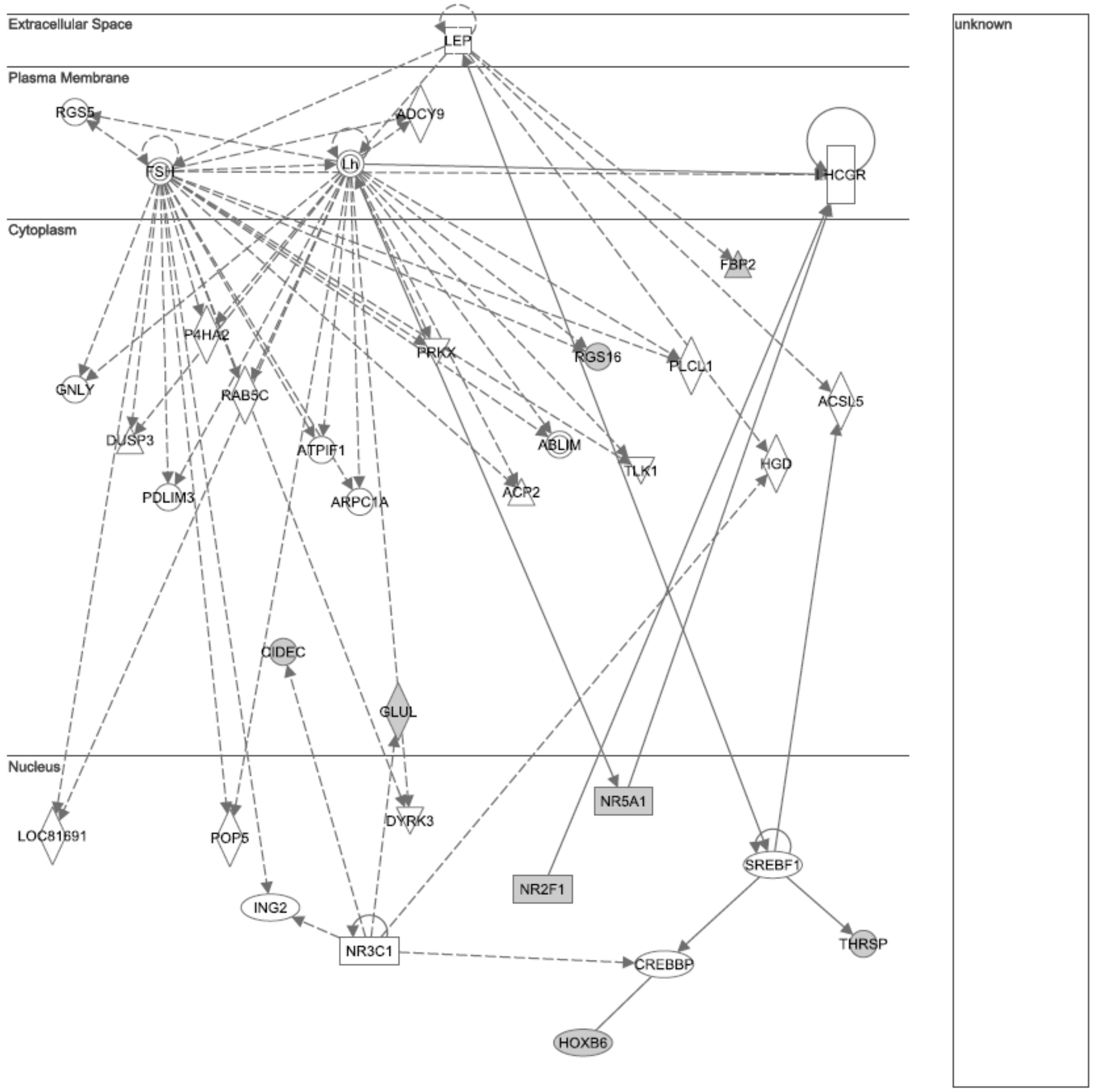

Supplementary Fig. 2 The top 3 # network significantly differentially expressed genes involved in. Solid line represents direct interaction and dash line represents indirect interaction
