# Supplementary material for: Functional Genomic Analysis of Variation on Beef Tenderness Induced by Acute Stress in Angus Cattle
Source: Comp Funct Genomics. 2012 Apr 12;2012:756284. doi: 10.1155/2012/756284 (PMC3332163; doi:10.1155/2012/756284)
Supplement: Supplementary file 3 [file 756284.f3.pdf]

Supplementary Table 1 Primers for RT-PCR

Genes	ProbeName	Accession Number	Primers	Sequence (5’ to 3’)	Size (bp)
HSPA1A	A_73_115519	NM_203322. 2	F	TCAACATGAAGAGCGCCGTGGA	114
			R	TGTTGGCGTCCAGCCAGGAAAT	
CXCL1	A_73_120022	NM_175700. 1	F	AGACTGGTCAGGAAGTGTGT	135
			R	CACTGAGGCTGCTGGAGTAT	
IL12A	A_73_119429	NM_174355. 1	F	AGACCAGAACATGCTGGCAG	142
			R	GAAGGCGTGAAGAAGGATGC	
JOSD1	A_73_120090	NM_001014898.2	F	GCCTTCACCCGGGAAACGCT	182
			R	ATGGCGCCAACATCCCTGCG	
GAPDH		NM_001034034. 1	F	GATTGTCAGCAATGCCTCCT	94
			R	GGTCATAAGTCCCTCCACGA	
