# Supplementary material for: Functional Genomic Analysis of Variation on Beef Tenderness Induced by Acute Stress in Angus Cattle
Source: Comp Funct Genomics. 2012 Apr 12;2012:756284. doi: 10.1155/2012/756284 (PMC3332163; doi:10.1155/2012/756284)
Supplement: Supplementary file 4 [file 756284.f4.pdf]

Supplementary Table 2 Primers for bisulfited-PCR

Gene name	ProbeName in Microarray	GeneBank Accession Number	Primers	Sequence (5' to 3')	Size (bp)
IL12A	A_73_119429	NM_174355. 1	F	AGAGGATGTTTTATAGGGYGTAG	167
			R	CRCTTCACAATAAATAACCAACC	
			S	ATAGGGYGTAGGATAAGT	
HSPA1A	A_73_115519	NM_203322. 2	F	GGATATYGTTTGYGTAGTTTGGTT	138
			R	TATTACCAACCCATTCCAATCACA	
			S	AGTTATAGGGGTTTTTTATT	
AP2B1	A_73_100724	NM_001075125. 1	F	TGTTAGGGATAAAGATGGTGGAT	103
			R	CAAAAAATAATCCCTCCTTTCT	
			S	AAAATAATCCCTCCTTTCT	
ASPM	A_73_107807	XM_614763. 5	F	GGGGTTGTTTAGGATTAGGG	97
			R	CCTATCCCTCAACCACTTCTACA	
			S	CCTCAACCACTTCTACAAAT	
DEPDC6	A_73_107834	XM_870049. 1	F	GGTGATTGTGATGGGTTTAGTTAT	143
			R	CCAAAAAAACAACCTCACCTCTA	
			S	CCCCCTACCCCATACT	
LOC614805	A_73_117241	XM_864993. 3	F	GTGGGGAAAAAATTTAAGATATT	145
			R	TAAATCCCCCAAATAAACCTCC	
			S	ATTAGGATTTAATTTGGTAG	

F: forward primer. R: reverse primer. S: sequencing. Y and R stand for C/T and G/A, respectively.
